# Blocking retrograde axonal transport of autophagosomes contributes to sevoflurane-induced neuron apoptosis in APP/PS1 mice

**DOI:** 10.1007/s13760-020-01359-6

**Published:** 2020-05-08

**Authors:** Mingliang Xu, Jianguo Feng, Mingxi Tang, Qingxi Guo, Jian Zhan, Fuzu Zhu, Hong Lei, Qingmei Kang

**Affiliations:** 1 Department of Anesthesiology, The Affiliated Hospital of Southwest Medical University, Luzhou, Sichuan People’s Republic of China; 2 Department of Pathology, The Affiliated Hospital of Southwest Medical University, Luzhou, Sichuan People’s Republic of China

**Keywords:** Alzheimer’s disease, Autophagy, Axonal transport, Sevoflurane

## Abstract

Autophagy, a crucial pathway for the degradation of proteins in eukaryotic cells, is linked to the development of Alzheimer’s disease (AD), and the accumulated autophagosomes in the cells resulting in the death of cells. Sevoflurane can impair spatial learning and memory in mice with AD and lead to the apoptosis of nerve cells; however, the underlying mechanisms remain unknown. We aim to explore the effects and underlying mechanisms of sevoflurane in APPswe/PS1ΔE9 double-transgenic mice. 51 heterozygous APPswe/PS1ΔE9 double-transgenic mice were involved and divided into three groups, including control group, sham group and sevoflurane group. Morris water maze experiment was used to test the learning and memory abilities of mice, flow cytometry was conducted to detect apoptosis and mitochondrial membrane potential of brain cells in mice, transmission electron microscopy was used to observe the number of autophagosomes at the axon in mice, and western blot was carried out to detect the expression of Bax, Bcl-2, LC3II, P62, KIF3B and DIC proteins of brain cells in mice. In our study, we found that significantly longer escape latencies, fewer crossings of the platform and shorter time spent in the target quadrant of the morris water maze experiment in the sevoflurane group. Flow cytometry showed cellular apoptosis was increased and the membrane potential of the mitochondria was reduced of brain cells in the sevoflurane group. Transmission electron microscopy displayed that there was a remarkable upregulation of autophagosomes at the axon of brain cells in mice after treatment of sevoflurane. Western blot demonstrated that the expression of Bax, LC3II, P62 and KIF3B proteins were elevated, and the expression of Bcl-2 and DIC proteins were reduced in the sevoflurane group. Sevoflurane impaired acquisition learning and memory function, promoted the apoptosis of hippocampal neurons in APPswe/PS1ΔE9 double-transgenic mice, and the mechanism might be related to the activation of autophagy along with the disruption of autophagosomes retrograde transport in axons.

## Introduction

As of 2015, approximately 900 million individuals in the world are aged over 60 years, and the percentage of Alzheimer’s disease (AD) patients is about 5–7% [1]. With an increase in the elderly population worldwide, more AD patients require surgical treatment and general anesthesia. With the advantages of strong controllability, rapid onset, and fewer side effects, inhalable anesthetics including isoflurane, sevoflurane, and desflurane are widely used for general anesthesia. However, growing evidence has demonstrated that inhalable anesthetics may be a risk factor for the subsequent development of postoperative cognitive dysfunction (POCD) and AD [2, 3]. Although several studies have revealed that sevoflurane, the most widely used inhalable anesthetic, can impair spatial learning and memory in AD transgenic mice [4, 5], some studies report a different conclusion suggesting that general anesthesia may not be associated with the development of AD [6]. Therefore, more evidence is needed to explore the effect of inhalable anesthetics on AD.

Macroautophagy hereafter referred to as autophagy, plays an important role in maintaining the stability of the intracellular environment and is a significant metabolic pathway in eukaryotic cells. Previous studies have shown that autophagy is implicated in various neurodegenerative diseases, including AD [7]. Further, autophagosomes associated with Amyloid β (Aβ) proteins exist at axonal terminals in the brains both mutant hAPP mice and AD patients, and a previous study has reported that autophagic dysfunction occurs in the brains both mutant human amyloid protein precursors (hAPP) mice and AD patients [8]. Autophagy can degrade abnormally folded proteins to exert a protective effect on nerve cells. However, if autophagosomes cannot be degraded successfully, the accumulation of them will lead to autophagic stress and the death of nerve cells [9, 10]. Zhang et al. [11] have reported that autophagy is involved in sevoflurane-induced cognitive dysfunction in aged rats. However, the role of autophagy in the sevoflurane-induced neuron apoptosis in APP/PS1 (amyloid precursor protein/presenilin 1) mice is unclear. Further, whether autophagic stress occurs in APP/PS1 mice after sevoflurane treatment is unknown.

One of the most important factors resulting in autophagic stress is a disruption in the formation of autolysosomes. Autophagosomes, produced at the axonal terminals, fuse with late endosomes and become amphisomes; these amphisomes then fuse with mature lysosomes to form autolysosomes after being transported to soma [12, 13]. This is a crucial step during degradation. During the axonal transport of autophagosomes, kinesin-mediated anterograde transport and dynein-mediated retrograde transport work together. Tammineni et al. [8] have revealed that the impairment of retrograde transport of axonal autophagosomes contributes to autophagic stress in AD neurons. However, whether treatment with sevoflurane results in changes in the retrograde transport in APP/PS1 mice remains unknown. Therefore, this study aimed to verify whether autophagic stress induces neuron apoptosis and cognitive impairment in APP/PS1 mice in response to sevoflurane treatment, and to explore the underlying mechanism.

## Materials and methods

### Animals

In this study, a total of 51 heterozygous APPswe/PS1ΔE9 double-transgenic mice (3 months old), which were widely used, were purchased from Beijing Huafukang Bioscience Co.Inc. (China). All mice were individually housed in ventilated cages with free access to water and food. The room temperature and humidity were maintained at 22 °C and 40–60%, respectively, with a 12-h light/12-h dark cycle. All animal experiments were approved by Institutional Animal Care and Use Committee of Southwest Medical University under the NIH Guide for the Care and Use of Laboratory Animals.

### Sevoflurane exposure

In all, 45 mice were randomly divided into three groups (*n* = 15 per group): control group, sham group, and sevoflurane group. Mice in the control group were placed in a chamber with no special intervention, whereas those in the sham group received humidified 100% O_2_ carrier gas for 2 h in an anesthetic induction chamber and the sevoflurane group mice were exposed to 2.5% sevoflurane (Hengrui Pharma, China) delivered via humidified 100% O_2_ carrier gas for 2 h in an identical chamber. The anesthetic induction chamber was connected to an anesthesia machine (Drager, Germany) through a threaded tube on one side, and it was connected to a threaded tube as an exhaust port on the other side. At the exhaust port, the concentration of sevoflurane in the anesthetic induction chamber was monitored using a multi-function gas monitor (Ge Medical Systems Information Technologies, Inc. USA). During the anesthesia process, warming mats were used to heat and maintain rectal temperatures of the mice at 37 ± 0.5 °C. Further, arterial oxygen, PH, and carbon dioxide tension were analyzed using a blood gas analyzer (Abbott Point of Care Inc., USA).

### Morris water maze

Twenty-four hours after treatment, the behaviors of eight mice per group were tested by the Morris water maze experiment. Morris water maze in this study was a circular tank (100 cm in diameter and 50 cm in height) filled with water to a height of 30 cm, with a white escape platform (9 cm in diameter and 1 cm below the water surface) in the middle of the third quadrant of the tank. The temperature of the water was maintained at 25 ± 0.5 °C.The test consisted of a 1-day visible platform test (1 cm above the water surface), 4-day learning and memory training, and spatial exploration experiment on the 6th day. During the second to 5th days, the mice were randomly placed in the water from each quadrant of the tank, and they had 60 s to find the platform. If they successfully find the platform within 60 s, the test was terminated and they were allowed to stay on the platform for 10 s to familiarize themselves with the environment. If they did not find the platform within 60 s, they were guided to the platform and kept there for 10 s. On the 6th day, the platform was removed and the spatial exploration experiment started. In this procedure, the mice swam freely for 60 s in the water and their escape latency, the number of times they crossed the platform, and the percentage of path length in the third quadrant were measured by the Morris water maze software on the computer.

### Tissue preparation

Further, 24 h after sevoflurane exposure, the mice in every group were sacrificed randomly. After anesthetizing the mice with 3.5% chloral hydrate (0.35 mg/g), the mice were transcardially perfused with phosphate-buffered saline (PBS, 1 ×). Following this, 4% paraformaldehyde and 2.5% glutaraldehyde were used to fix the hippocampi of four mice per group (two more mice per group were added later) for transmission electron microscopy (TEM). Half hippocampi of five mice were dissected promptly, frozen immediately in liquid nitrogen, and stored at – 80 °C for Western blotting (WB). The other hippocampi were used for flow cytometry test.

### Apoptosis and mitochondrial membrane potential measurements by flow cytometry

For the assessment of apoptosis and mitochondrial membrane potential of cells in the hippocampus (five mice per group), flow cytometry was performed. First, the hippocampi of the mice were cut, phosphate-buffered saline (PBS, 1 ×) was mixed, and the mixture was centrifuged at low speed (Thermo Fisher Scientific, 500 rpm) for 3 min. Following this, trypsin (Gene View) was added for digestion and the mixture was maintained at 37 °C in a water bath for 30 min. Finally, cells were harvested for apoptosis and mitochondrial membrane potential measurements after washing three times with ice-cold PBS.

For apoptosis analysis by flow cytometry, cells were incubated with a binding buffer containing AnnexinV-FITC and propidium iodide (PI, KeyGEN Biotech, Nanjing, China) for 30 min at 37 °C in the dark. Following this, the cells were washed three times and resuspended in the binding buffer (200 μl). Following this, they were analyzed by flow cytometry, and early apoptotic (in the fourth quadrant, Annexin V + /PI–) and late apoptotic (in the first quadrant, Annexin V + /PI +) cells were identified.

For the evaluation of mitochondrial membrane potential of cells by flow cytometry, cells were stained with 5 μg/ml Rhodamine 123 (Sigma-Aldrich, USA) and incubated at 37 °C for 30 min in the dark. After washing three times and resuspension in 47% Dulbecco’s Modified Eagle Medium (DMEM, 1 × , Gibco, 200 μl), the fluorescence intensities (excitation 532 nm; emission 585 nm) of cells were measured using a FACSArray bioanalyzer (BD Biosciences).

### Transmission electron microscopy

For TEM, the hippocampi of four mice per group were cut into 1 mm^3^ tissue sections. After fixing with glutaraldehyde for 2 h, the tissues were washed with PBS (0.01 M) and post-fixed in osmium tetroxide (1%) for 2 h. Following this, a gradient series of alcohol solutions was added to dehydrate. Then, the tissues were embedded in epoxy resin (Epon 812), cut into semithin sections (1 μm) and stained with azure-methylene blue. After examination under a light microscope, the key sections of the semithin sections were cut again (120 nm). At last, these thin sections were stained with uranyl acetate (4%) and lead citrate (0.5%). Autophagosomes in the neurons of the hippocampus were observed by TEM (Philips EM208S).

### Western blot assay

Proteins in the hippocampus and cortex (five mice per group) were lysed using radioimmunoprecipitation assay (RIPA, Beyotime) lysis buffer and phenylmethanesulfonyl fluoride (PMSF, Beyotime) (RIPA:PMSF = 100:1) supplemented with protease inhibitors (Roche). After determination of protein concentration using BCA Protein Assay Kit (Beyotime), equal amounts of protein (50 μg) was separated by SDS-PAGE and electrotransferred onto polyvinylidene fluoride (PVDF) membranes (Millipore, Billerica, MS, USA). Then the PVDF membranes were blocked using 5% nonfat milk for 2 h, incubated with primary antibodies (4 °C) overnight, and incubated with HRP-conjugated secondary anti-rabbit or anti-mouse antibodies (1:5000, Multi Sciences, China) for 1 h at 37 °C. Immunoreactive bands were observed by enhanced chemiluminescent substrate (ECL, Pierce) exposure to X-ray films. At last, the bands were quantified using Image J, and the relative intensity of each band was normalized to the band of β-actin.

Target proteins were detected using the following primary antibodies: Bax (Abcam, 1:1000), Bcl-2 (Abcam, 1:500), LC3 (Sigma-Aldrich, 1:1000), P62 (Cell Signaling Technology, 1:1000), DIC (Millipore, 1:1000), KIF3B (Cell Signaling Technology, 1:1000), and β-actin (Beijing 4A Biotech Co., Ltd, 1:5000).

### Statistical analysis

All data were analyzed using SPSS 18.0 statistical software and presented as means ± SD from three independent experiments at least. A standard two-tailed unpaired *t* test was performed to analyze the comparison between two groups and one-way ANOVA was performed to evaluate multiple comparisons. For the analysis, *P* < 0.05 was regarded as significant throughout.

## Results

### Sevoflurane resulted in the impairment of learning and memory in APP/PS1 mice

**Fig. 1:**
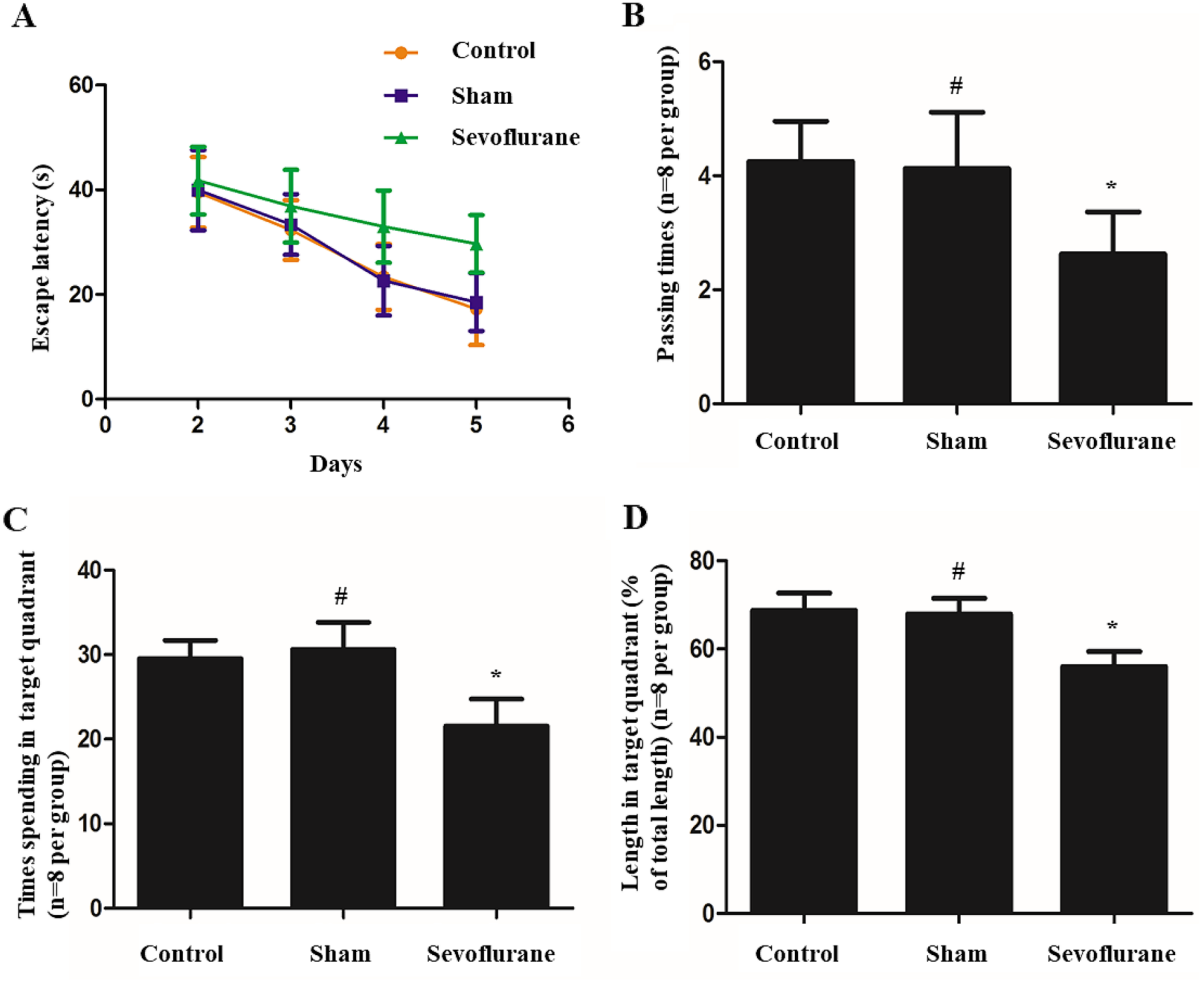
Morris water maze analysis showed spatial learning and memory in three groups. **a** Graph above showed the escape latency was gradually reducing in relation to the number of days of training. Significantly longer escape latency time was performed in the Sevoflurane group. **b** The platform-passing times of mice in the probe test. **c** The time spending in the target quadrant in the probe test. **d** The length (%) spent in the target quadrant in the probe test. (*P < 0.01, vs. Control group; ^#^*P* > 0.05, vs. Control group, *n* = 8 per group)

During the spatial learning phase (when the platform in the target quadrant was visible), the escape latency gradually reduced in relation to the number of days of training. However, significantly longer escape latencies were observed in the sevoflurane group (*n* = 8), and the escape latencies were shorter in the control group (*n* = 8) and sham group (*n* = 8) (Fig. 1a). In the probe test (when the platform in the target quadrant was removed), the sevoflurane group showed fewer crossings of the platform and shorter time spent in the target quadrant compared with the control and sham groups. However, there was no significant difference between control and sham groups in this regard (Fig. 1b–d). Accordingly, there was cognitive impairment in APP/PS1 mice with sevoflurane exposure.

### Sevoflurane induced hippocampal neuron apoptosis in APP/PS1 mice

**Fig. 2:**
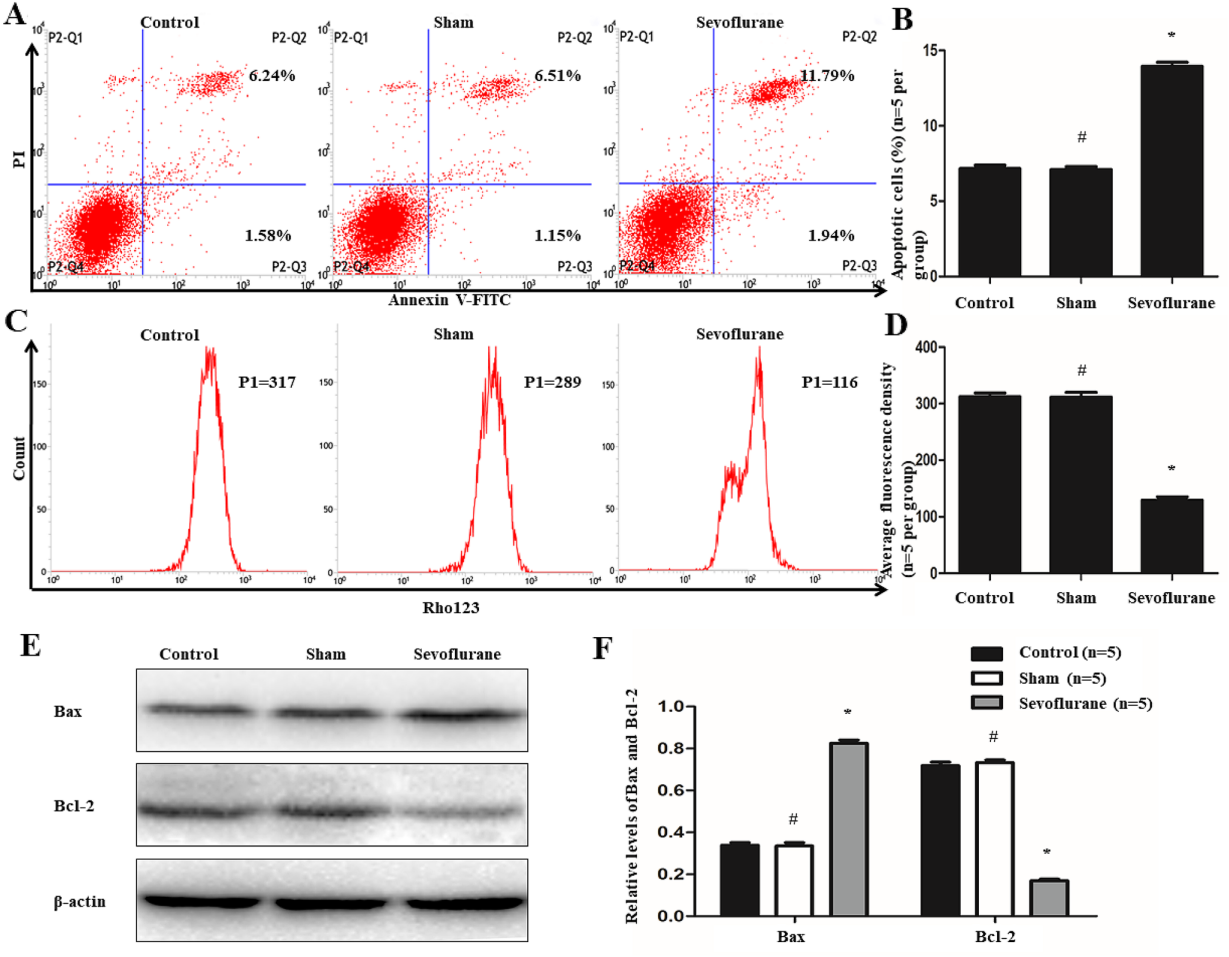
Effects of sevoflurane on the apoptosis of hippocampal neuron. **a** and **b** Flow cytometry was conducted to analysis apoptosis rates of cells, P2–Q2 (Annexin V + /PI +) and P2–Q3 (Annexin V + /PI−) represented the rates of late and early apoptosis of cells in (**a**), respectively. The results showed the total ratios of apoptotic cells were 7.15 ± 0.45% in Control group, 7.10 ± 0.39% in Sham group and 13.94 ± 0.55% in Sevoflurane group. Compared with the Control group, the apoptosis of cells were increased in Sevoflurane group but there were no significant changes in Sham group. **c** and **d** Flow cytometry was also used to determine the membrane potential of mitochondrial. And after treatment of sevoflurane, the fluorescence intensity of Rhodamine 123 was remarkably decreased. **e** and **f** Western blot was performed to test the levels of Bax and Bcl-2. The expression of Bax protein was elevated, while the level of Bcl-2 was attenuated after the treatment of sevoflurane. (*P < 0.01, vs Control group; ^#^*P* > 0.05, vs Control group, *n* = 5 per group)

Flow cytometry was conducted to detect apoptosis and mitochondrial membrane potential of cells that separated from the hippocampi of the mice. P2–Q2 (Annexin V + /PI +) and P2–Q3 (Annexin V + /PI–) represented the rates of late and early apoptosis of cells in Fig. 2a, respectively. There was no significant change in cellular apoptosis between the sham and control groups (7.10 ± 0.39% vs. 7.15 ± 0.45%, *P* > 0.05, *n* = 5 per group) (Fig. 2a and b). However, compared with the control group, cellular apoptosis was increased in the sevoflurane group (13.94 ± 0.55% vs. 7.15 ± 0.45%, *P* < 0.01, *n* = 5 per group) (Fig. 2a and b). Furthermore, in this experiment, we also found that after treatment of sevoflurane, not only late apoptotic but also early apoptotic of the cells were increased (11.90 ± 0.47% vs. 5.86 ± 0.27%, *P* < 0.01, *n* = 5 per group; 2.04 ± 0.12% vs. 1.30 ± 0.23%, *P* < 0.01, *n* = 5 per group). And there were still no significant alterations in late and early apoptotic of the cells between sham and control groups (5.78 ± 0.50% vs. 5.86 ± 0.27%, *P* > 0.05, *n* = 5 per group; 1.31 ± 0.26% vs. 1.30 ± 0.23%, *P* > 0.05, *n* = 5 per group) (Fig. 2a). Meanwhile, Rhodamine 123 was used as a fluorescent dye to detect the mitochondrial membrane potential of cells because it can across the membrane and be accumulated in the mitochondria. As shown in Fig. 2c and d, the fluorescence intensity of Rhodamine 123 was remarkably weaker in the sevoflurane group than in the control group (129.50 ± 11.90 vs. 312.00 ± 13.61, *P* < 0.01, *n* = 5 per group), whereas there was no significant difference in the intensities between sham and control groups (311.50 ± 16.90 vs. 312.00 ± 13.61, *P* > 0.05, *n* = 5 per group).

In addition, the levels of Bax and Bcl-2 were examined by Western blot analysis. The results demonstrated that Bax expression was elevated (0.83 ± 0.03 vs. 0.34 ± 0.02, *P* < 0.01, *n* = 5 per group) but Bcl-2 expression was reduced (0.17 ± 0.02 vs. 0.72 ± 0.03, *P* < 0.01, *n* = 5 per group) in the sevoflurane group, which compared with Control group (Fig. 2e and f). Furthermore, compared with the control group, the expression of Bax (0.34 ± 0.03 vs. 0.34 ± 0.02, *P* > 0.05, *n* = 5 per group) and Bcl-2 (0.73 ± 0.02 vs. 0.72 ± 0.03, *P* > 0.05, *n* = 5 per group) proteins did not change significantly in the Sham group.

### Sevoflurane accelerated autophagy and blocked autophagy flux in APP/PS1 mice

**Fig. 3:**
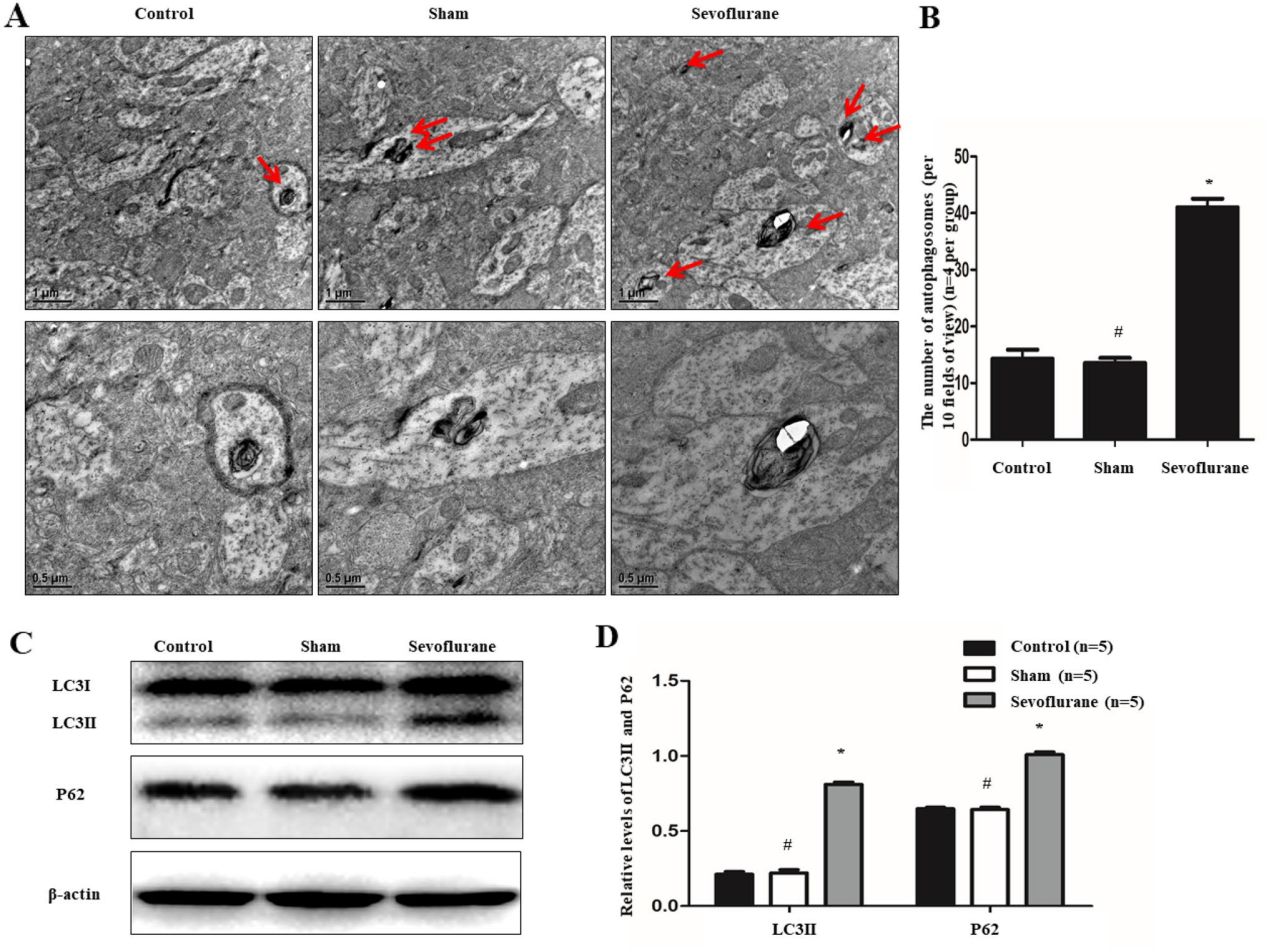
Effects of sevoflurane on autophagy and autophagic flux of APP/PS1 mice. **a** and **b** Transmission electron microscope was employed to analyze the number of autophagosomes. And the data displayed that there was a remarkable upregulation of autophagosomes at the distal axon after treatment of sevoflurane (*n* = 4 per group). **c** and **d** Furthermore, western blot analysis showed sevoflurane could not only enhance the levels of LC3II but also increase the expression of P62 (*n* = 5 per group). (*P < 0.01, vs. Control group; ^#^*P* > 0.05, vs. Control group)

Double membrane vacuolar structures called autophagosomes were observed by TEM. Ten fields of vision were randomly selected from the control, sham, and sevoflurane groups to calculate the number of autophagosomes. As shown in Fig. 3a and b, the data displayed that there was a remarkable upregulation of autophagosomes at the axon in the sevoflurane group, compared with the control group (40.67 ± 5.13 vs. 15.00 ± 3.61, *P* < 0.01, *n* = 4 per group). However, there was no significant difference in the number of autophagosomes at the axon between the sham group and control group (14.33 ± 1.15% vs. 15.00 ± 3.61, *P* > 0.05, *n* = 4 per group).

Furthermore, Western blot was performed to detect the expression of autophagy-related proteins, including those encoding LC3II and P62 proteins. The result displayed that compared with the level of LC3II protein in the control group, that in the sevoflurane group was enhanced (0.81 ± 0.03 vs. 0.21 ± 0.03, *P* < 0.01, *n* = 5 per group) and that in the sham group was similar (0.22 ± 0.04 vs. 0.21 ± 0.03, *P* > 0.05, *n* = 5 per group) (Fig. 3c and d). Meanwhile, the same result was found for the expression of P62 protein. Compared with the expression of P62 protein in the control group, the expression of it in the sevoflurane group was upregulated (1.01 ± 0.04 vs. 0.65 ± 0.02, *P* < 0.01, *n* = 5 per group) and that in Sham group was identical (0.64 ± 0.03 vs. 0.65 ± 0.02, *P* > 0.05, *n* = 5 per group) (Fig. 3c and d).

### Sevoflurane inhibited the retrograde axonal transport of autophagosomes in APP/PS1 mice

**Fig. 4:**
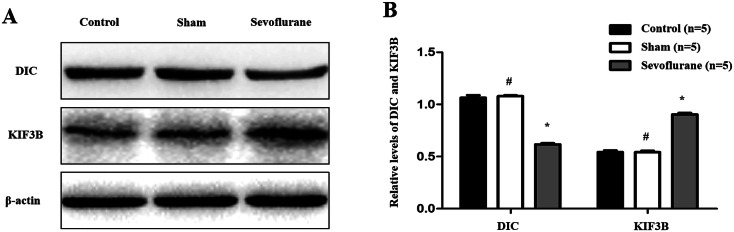
Effects of sevoflurane on axonal transport of autophagosomes in APP/PS1 mice. **a** and **b** Western blot was employed to determine the expression of DIC and KIF3B proteins. As shown above, the expression of DIC protein in Sevoflurance group was lower than that in the Control group, but the expression of KIF3B protein was higher in Sevoflurance group than that in the Control group. (*P < 0.01, vs. Control group; ^#^*P* > 0.05, vs. Control group, *n* = 5 per group)

Western blot analysis was employed to determine the expression of dynein intermediate chain (DIC) protein, mainly responsible for retrograde axonal transport, and kinesin family member 3B (KIF3B) protein, mainly responsible for the antegrade axonal transport. As shown in Fig. 4a and b, the expression of DIC protein in the sevoflurane group was lower than that in the control group (0.62 ± 0.02 vs. 1.06 ± 0.05, *P* < 0.01, *n* = 5 per group). Conversely, the opposite result was shown for the expression of KIF3B protein, which was higher in the sevoflurane group than that in the control group (0.90 ± 0.03 vs. 0.54 ± 0.03, *P* < 0.01, *n* = 5 per group). However, there were no significant differences in the levels of DIC (1.08 ± 0.02 vs. 1.06 ± 0.05, *P* > 0.05, *n* = 5 per group) and KIF3B (0.54 ± 0.03 vs. 0.54 ± 0.03, *P* > 0.05, *n* = 5 per group) between the sham group and the control group.

## Discussion

In the previous studies, the effect of sevoflurane, the most commonly used inhalable anesthetic, on AD has been controversial. Although researchers have found that sevoflurane can impair spatial learning and memory in AD transgenic mice [4, 5], other studies have demonstrated that anesthesia may not contribute to the development of AD [14, 15]. Our results showed that after treatment with sevoflurane, spatial learning and memory of APP/PS1 mice were decreased. To find the reason behind this learning and memory deficit, we further detected the state of hippocampal neurons. In the early apoptotic cells, the membrane potential of the mitochondria reduced and the ability to aggregate dyes also decreased. Therefore, we can determine early apoptosis of cells by measuring the intensity of fluorescence by flow cytometry after staining the cells with suitable dyes (such as Rhodamine 123, Chloroethyl red, and JC-1). Lu et al. revealed that sevoflurane-induced caspase activation and apoptosis in AD transgenic mice [16]. In our study, the rate of cellular apoptosis was elevated, mitochondrial membrane potential of cells was declined, the expression of the pro-apoptotic protein Bax was upregulated, and the level of the anti-apoptotic protein Bcl-2 was reduced in response to sevoflurane exposure. All results suggested that sevoflurane specifically induces apoptosis in hippocampal neurons, leading to the deficit of learning and memory in APP/PS1 mice. However, the underlying mechanism is unknown.

Cellular apoptosis mechanism is complex and is involved in pathways, such as the death receptor-mediated pathway, the mitochondrial pathway, and the endoplasmic reticulum (ER) stress-mediated pathway. However, other ways, including autophagy, have been getting more attention. Autophagy is a lysosomal degradation process. In this process, abnormally folded proteins, such as Aβ protein, implicated in the pathology of AD, are encapsulated within autophagosomes before being degraded by lysosomal hydrolases in autolysosomes [8]. Zhang et al. [11] reported that impaired autophagy in the hippocampal neurons of aged rats after sevoflurane treatment may contribute to hippocampal neuron apoptosis and cognitive impairment. Further evidence is required to discover the relationship between autophagy and AD after sevoflurane treatment. Our data demonstrated that autophagy was activated, autophagosomes were stacked at the axon, but autophagy flux was blocked after sevoflurane treatment. This was the key means resulting in cellular apoptosis. In contrast to our results, Zhou et al. [17] found that sevoflurane-induced ER stress and activated autophagy to antagonize sevoflurane-induced apoptosis in H4 human neuroglioma cells. However, consistent with our results, Geng et al. [5] clarified that autophagic degradation deficit was involved in sevoflurane-induced amyloid pathology in APP/PS1 transgenic mice. Unlike the study by Geng et al., our experiments suggest that autophagic degradation deficit may cause neuronal apoptosis in the hippocampus after sevoflurane treatment. We further explored the mechanism of the degradation disorder in autophagosomes.

Transporting autophagosomes containing engulfed damaged organelles and aggregated proteins from distal axons, where they are continuously generated, to the soma, where mature acidic lysosomes are mainly located, is challenging [8]. Studies have provided evidence suggesting that the movement of autophagosomes is largely blocked by the dynein motor inhibitor [18], and the retrograde transport of the autophagosome-containing Aβ oligomers in axons is closely associated with dynein [8]. Dynein intermediate chain (DIC), a key part of the dynein complex, plays a role in cargo binding and is involved in retrograde transport of cargo specificity, which is important for the combination of autophagosomes and lysosomes. Interactions of DIC and dynactin complex can modulate the movement of dynein. Further, DIC may also directly interact with protein cargos, such as huntingtin, casein kinase II, and β-catenin [19]. In previous studies, it has been demonstrated that DIC is required for the retrograde transport of vesicular cargos along microtubules and is beneficial for aggregated proteins being transported to juxtanuclear positions for autophagic clearance [20, 21]. In our study, we found that sevoflurane could suppress DIC expression. KIF3B, a member of kinesin superfamily, can bind to KIF3A and kinesin superfamily-associated protein 3 (KAP3) to form a complex, which demonstrates plus end-directed microtubule-sliding activity and is a key factor for the maturation of autophagosomes in vitro [22]. From our results, we found that sevoflurane can elevate the level of KIF3B protein. These findings indicate that sevoflurane is beneficial to the anterograde transport in axons for promoting the maturation of autophagosomes, while it is deficient in retrograde transport in axons to suppress the integration of autolysosomes. Of course, regulation of axonal transport occurs at multiple levels including modulation of the microtubule track, cargo-specific adaptors, and scaffolding proteins that coordinate cargo-bound motors. Therefore, more factors should be considered in our next study.

In conclusion, our study provided evidence suggesting that sevoflurane impairs spatial learning and memory in APP/PS1 mice and elevates the apoptosis rates of hippocampal neurons by increasing the activation of autophagy while blocking autophagic flux. The mechanism may be related to the damage of retrograde axonal transport of autophagosomes. Thus, our findings may provide novel ideas and therapeutic targets for AD. However, the process of axonal transport of autophagosomes is complex and many factors are involved, such as the transport of proteins, RNA, and organelles over long distances require molecular motors that operate along the cellular cytoskeleton. Further research is required to find the reason for deficit of axonal transport.
